# External validation of clinical prediction models using big datasets from e-health records or IPD meta-analysis: opportunities and challenges

**DOI:** 10.1136/bmj.i3140

**Published:** 2016-06-22

**Authors:** Richard D Riley, Joie Ensor, Kym I E Snell, Thomas P A Debray, Doug G Altman, Karel G M Moons, Gary S Collins

**Affiliations:** 1 Research Institute for Primary Care and Health Sciences, Keele University, Keele ST5 5BG, Staffordshire, UK; 2 Institute of Applied Health Research, University of Birmingham, Edgbaston, Birmingham, UK; 3 Julius Centre for Health Sciences and Primary Care, University Medical Center Utrecht, Utrecht, Netherlands; 4 Cochrane Netherlands, University Medical Center Utrecht, Utrecht, Netherlands; 5 Centre for Statistics in Medicine, Nuffield Department of Orthopaedics, Rheumatology and Musculoskeletal Sciences, University of Oxford, Oxford, UK

## Abstract

Access to big datasets from e-health records and individual participant data (IPD) meta-analysis is signalling a new advent of external validation studies for clinical prediction models. In this article, the authors illustrate novel opportunities for external validation in big, combined datasets, while drawing attention to methodological challenges and reporting issues.

**Summary points**

Clinical prediction models are used to predict the risk of disease presence and outcome occurrence in individuals, thereby informing clinical diagnosis and prognosis
Increasingly, researchers undertaking prediction model research have access to so-called “big” datasets from meta-analyses of individual participant data (IPD), or registry databases containing electronic health records for thousands or even millions of patients
Such big datasets heralds an exciting opportunity to improve the uptake and scope of external validation research, to check whether a model’s predictions are reliable
In particular, they allow researchers to externally validate a model’s predictive performance (eg, in terms of calibration and discrimination) across all clinical settings, populations, and subgroups of interest
If a model has poor predictive performance, big datasets help identify if and how updating or tailoring strategies (such as recalibration) can improve performance for particular settings, clusters or subgroups (rather than simply discarding the model)
However, big datasets may also bring additional methodological challenges and reporting criteria

****A popular type of clinical research is the development of statistical models that predict disease presence and outcome occurrence in individuals,^1^ ^2^ ^3^ thereby informing clinical diagnosis and prognosis. Such models are referred to here as diagnostic and prognostic prediction models, but they have many other names including risk models, risk scores, and clinical prediction rules. They are typically developed by use of a multivariable regression framework, which provides an equation to estimate an individual’s risk based on values of multiple predictors (such as age and smoking, or biomarkers and genetic information). Figure 1 gives the format of equations based on logistic or Cox regression, which involve an intercept or baseline hazard term combined with multiple predictor effects (corresponding to odds or hazard ratios). Well known examples are the Framingham risk score and QRISK2,^4^ ^5^ which estimate the 10 year risk of developing cardiovascular disease; the Nottingham prognostic index, which predicts the five year survival probability of a woman with newly diagnosed breast cancer^6^ ^7^; and the Wells score for predicting the presence of a pulmonary embolism.^8^ ^9^

**Fig 1 f1:**
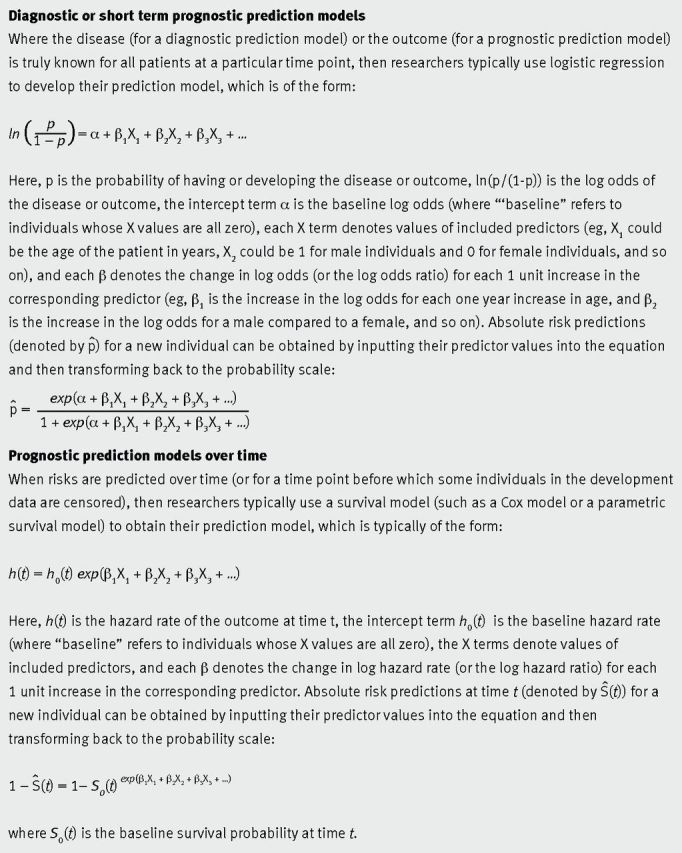
Format of typical prediction models seen in the medical literature

In 2009, *The BMJ* published a series of four articles to guide those undertaking prediction model research,^2^ ^10^ ^11^ ^12^ and further recommendations were made in the 2013 PROGRESS series.^3^ ^13^ ^14^ ^15^ These articles all emphasised three fundamental components of prediction model research: model development, external validation, and impact evaluation. 

Model development is the process that leads to the final prediction equation, and involves many aspects detailed elsewhere.^2^ ^16^ ^17^ ^18^ Impact studies evaluate, ideally in a randomised trial, whether the implementation of a prediction model in clinical practice actually improves patient outcomes by informing treatment decisions according to the model’s predicted risk. However, impact studies should not be considered until the robustness and generalisability of a developed model is verified in one or more external validation studies.^3^ ^19^

External validation uses new participant level data, external to those used for model development, to examine whether the model’s predictions are reliable (that is, accurate enough) in individuals from potential population(s) for clinical use.^20^ Unfortunately, most prediction research focuses on model development and there are relatively few external validation studies.^3^ ^21^ ^22^ ^23^ This leads to a plethora of proposed models, with little evidence about which are reliable and under what circumstances. Confusion then ensues: promising models are often quickly forgotten,^24^ and—of more concern—many models may be used or advocated without appropriate examination of their performance.^25^

A shortage of external validation studies is often attributed to the lack of data available besides those data used for model development. Data from one study (eg, a cohort study) usually have a limited number of events. Hence all data are best retained for model development, rather than splitting the data so that a part is used for development and the remainder for validation.^26^ However, increasingly researchers have access to “big” data, as evident by meta-analyses using individual participant data (IPD) from multiple studies,^27^ ^28^ ^29^ ^30^ and by analyses of registry databases containing electronic health (e-health) records for thousands or even millions of patients from multiple practices, hospitals, or countries.^31^

For example, QRISK2 was developed by use of e-health data from the QRESEARCH database. The database uses over 1.5 million patients (with over 95 000 new cardiovascular events) from 355 randomly selected general practices,^5^ with external validation carried out by independent investigators in an additional 1.6 million patients from another 365 practices.^32^ In the IPD meta-analysis setting, an example is the IMPACT consortium, which developed a prediction model for mortality and unfavourable outcome in traumatic brain injury. The consortium shared IPD from 11 studies (8509 patients), and performed external validation using IPD from another large study (6681 patients).^33^

Such big, combined datasets heralds an exciting opportunity to improve the uptake of external validation research. Here, we describe the additional opportunities, challenges, and reporting issues involved in prediction research in this situation. We begin by introducing two key performance measures (calibration and discrimination) and a review of current practice in external validation research. Then, using five empirical examples, we show how big datasets allow a model’s predictive performance to be more fully interrogated across different populations, subgroups, and settings. We conclude by signposting methodological challenges and reporting criteria, which build on the recent TRIPOD statement for the transparent reporting of a multivariable prediction model for individual prognosis or diagnosis.^34^ ^35^

## Predictive performance of a model in terms of discrimination and calibration

External validation of a prediction model typically involves quantifying a model’s discrimination and calibration performance in data that were not used to develop the model. To be useful, a model’s predicted risks must discriminate (separate) well between those participants who do and do not have the outcome (disease or event) of interest. Discrimination is usually measured by the C statistic,^18^ and for survival outcomes also the D statistic (box 1).^36^ Calibration examines the agreement between predicted and observed risks, and can be quantified by measures such as the calibration slope and the expected/observed (E/O) statistic (box 1). Calibration can also be visualised graphically, for example, by plotting observed versus predicted risks across tenths of predicted risk,^10^ using a flexible calibration plot with a smoothed non-linear curve generated using a loess smoother or splines,^10^ ^37^ or displaying observed and predicted survival curves over time for different risk groups.^38^

**Box 1: Key measures for calibration and discrimination**

**Calibration slope**

For a perfectly calibrated model, we expect to see that, in 100 individuals with a predicted risk of r% from our model, r of the 100 truly have the disease (for diagnostic prediction) or outcome (for prognostic prediction) of interest. The calibration slope is one measure of agreement between observed and predicted risk of the event (outcome) across the whole range of predicted values,^1^ ^18^ and should ideally be 1.

A slope <1 indicates that some predictions are too extreme (eg, predictions close to 1 are too high, and predictions close to 0 are too low), and a slope >1 indicates predictions are too narrow. A calibration slope <1 is often observed in validation studies, consistent with over-fitting in the original model development.

**Expected/observed number of events (E/O)**

E/O summarises the overall calibration of risk predictions from the model in the entire validation sample (it is closely related to the so-called “calibration in the large,”^1^ but more intuitive to interpret). It provides the ratio of the total expected to have disease (outcome) to the total observed with disease (or with outcome by a particular time point). Thus, an ideal value is 1. Values less than 1 indicate the model is under-predicting the total number of events in the population, while values above 1 indicate it is over-predicting the total number events in the population.

Sometimes, in addition to looking at E/O across the entire dataset, E/O is reported for groups of predicted risk (for example, by tenths of predicted risk). The E/O ratios then describe the shape of the calibration slope. Note also that sometimes the O/E ratio is presented; under-prediction then occurs for values above 1 and over-prediction for values less than 1.

**C statistic**

The C statistic is a measure of a prediction model’s discrimination (separation) between those with or without the outcome. Also known as the concordance index or, for binary outcomes, the area under the receiver operating characteristic (ROC) curve. It gives the probability that for any randomly selected pair of individuals, one with and one without the disease (outcome), the model assigns a higher probability to the individual with the disease (outcome). A value of 1 indicates the model has perfect discrimination, while a value of 0.5 indicates the model discriminates no better than chance.

**D statistic**

The D statistic is a measure of discrimination for time-to-event outcomes only.^36^ This can be interpreted as the log hazard ratio comparing two equally sized groups defined by dichotomising at the median value of the prognostic index from the developed model (where the prognostic index is defined by the combined predictor effects in the developed model, (that is, β_1_X_1_+β_2_X_2_+β_3_X_3_+ . . . ). Higher values for the D statistic indicate greater discrimination. A related statistic is R^2^_D_.^36^

## Current shortcomings of external validation studies

A systematic review of 78 external validation studies published in 2010 concluded that “there is a dearth of well-conducted and clearly reported external validation studies.”^39^ Although model discrimination was usually reported, 68% of studies did not report evaluating model calibration, and only 11 (14%) presented a calibration plot. It was also often unclear how missing data were handled and even which model (the original model or some simplified version of it) was being evaluated. Further, sample size was often small, with 46% having fewer than 100 events, which is a minimum effective sample size suggested for external validation^40^ ^41^ (although an increase to 200 was recently proposed to assess calibration^37^ ^41^). Other reviews have identified similar problems.^21^ ^23^

A major problem of external validation studies is that they are often based on small and local datasets. For this reason, most external validation studies can, at best, assess the performance of a prediction model in a specific setting or population. However, it is increasingly recognised that the predictive performance of a model tends to vary across settings, populations and periods.^20^ ^30^ ^42^ ^43^ This implies that there is often heterogeneity in model performance, and that multiple external validation studies are needed to fully appreciate the generalisability of a prediction model.^20^ Although multiple datasets are increasingly available for this purpose,^29^ studies with access to such data mainly focus on model development and often ignore external validation.^28^ Hence, heterogeneity in model performance across populations, settings, and periods is rarely assessed. 

Similar deficiencies are apparent in external validation studies using big datasets from e-health records or disease registries. For example, after development of the QRISK2 model using routinely collected data from 355 primary care practices, Hippisley-Cox and colleagues^5^ immediately evaluated the model’s performance using further data from an additional 176 practices. However, potential heterogeneity in model performance across these 176 practices was ignored, with calibration and discrimination only summarised across all practices combined. Similarly, the independent external validation of QRISK2 by Collins and Altman^44^ ignored between-practice heterogeneity. Therefore, it remains unclear whether QRISK2 performs better or worse in some practices, regions, or (sub)populations than in others, and we return to this issue in examples 2 and 4 below.

## What causes heterogeneity in model performance?

There are several potential causes of heterogeneous model performance across different settings and populations,^29^ ^43^ ^45^ which can occur in isolation or in combination. A major reason is different case mix variation, which is similar to the “spectrum effect,”^46^ ^47^ a term used to describe variation in test accuracy performance across different populations and subgroups. Here “case mix” refers to the distribution of predictor values, other relevant participant or setting characteristics (such as treatment received), and the outcome prevalence (diagnosis) or incidence (prognosis). Case mix variation across different settings or populations can lead to genuine differences in the performance of a prediction model, even when the true (underlying) predictor effects are consistent (that is, when the effect of a particular predictor on outcome risk is the same regardless of the study population).^43^

It is, for instance, well known that the performance of models developed in secondary care is usually different when they are applied in a primary care setting, because the outcome prevalence or distribution of predictor values will be different.^48^ For example, the Wells score is a diagnostic prediction model for deep vein thrombosis, which was developed in secondary care outpatients. However, Oudega and colleagues^49^ show that it does not adequately rule out deep vein thrombosis in primary care patients, because 12% of patients in the low risk group had deep vein thrombosis compared with 3% in the original secondary care setting. The higher prevalence is due to a change in the selection and definition of patients with suspected deep vein thrombosis, leading to a different distribution of predictor values and case mix variation in primary care compared with secondary care.

The magnitude of predictor effects (denoted by β in fig 1) might also depend on the case mix itself. For example, in the cancer field, the effect of a biomarker may vary (interact) with particular subgroups, such as the stage of disease or the treatment received, and its relation with outcome risk might be non-linear. However, such interactions and non-linear trends are often missed (or mis-specified) when developing a model. Further, a biomarker is often measured differently (eg, by equipment from different manufacturers, or by a different assay or technique), recorded at a different time point (eg, before or after surgery), or quantified differently (eg, by a different cut-off point to define high and low values) across settings. Many other clinical, laboratory, and methodological differences can also exist, including differences in treatment strategies, clinical guidelines, and experience; disease and outcome definitions; and follow-up lengths. All these problems may lead to heterogeneity in predictor effects.^14^ ^50^ Subsequently, a developed model including predictor effects from one population might not perform well in a different population in which the magnitude of predictor effects are different because of the change in case mix, and use of different clinical, laboratory, and methodological standards.

Another key source is heterogeneity in the average prevalence (incidence) of the disease (outcome) to be predicted. This heterogeneity is caused, for example, by different standards of care and administered treatment strategies across regions and countries, and different starting points (eg, earlier diagnosis of disease in some populations due to a screening programme).^13^ This leads to differences across populations in the baseline risk, and thus the intercept (or baseline hazard rate; see fig 1) of a developed model might not be transportable from one population to another, leading to predicted risks that are systematically too low or too high. This is one reason for so-called “model updating,”^51^ where the intercept (baseline hazard) or predictor effects of a previous model are updated to recalibrate predictive performance to the new population.

## Opportunities to improve external validation using big data

Here, we use five empirical examples to demonstrate how big datasets from e-health records or IPD meta-analysis allow researchers to examine heterogeneity and (if necessary) improve the predictive performance of a model across different populations, settings, and subgroups. Examples 1 and 2 consider ways to investigate the extent of heterogeneity, whereas examples 3 to 5 examine the sources of heterogeneity and how to tailor (recalibrate) the model to the new circumstances.

### Example 1: Examining consistency in a model’s predictive performance across multiple studies

**Fig 2 f2:**
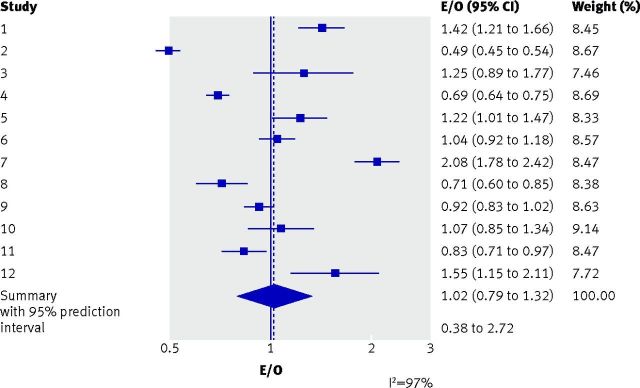
Calibration performance (as measured by the E/O statistic) of a diagnostic prediction model for deep vein thrombosis,^45^ over all studies combined and in each of the 12 studies separately. E=total number expected to have deep vein thrombosis according to the prediction model; O=total number observed with deep vein thrombosis; I^2^=proportion (%) of variability in the ln(E/O) estimates in the meta-analysis that is due to between-study variation (genuine differences between studies in the true ln(E/O)), rather than within-study sampling error (chance)

When data from multiple studies are available for external validation, meta-analysis techniques (such as a random effects meta-analysis^52^) can be used to quantify and summarise between-study heterogeneity in model performance.^30^ ^53^ ^54^ For example, Debray and colleagues developed a prediction model for the diagnosis of deep vein thrombosis in patients suspected of having the condition.^45^ The researchers performed external validation using 12 studies (10 014 patients in total; study sample sizes ranging from 153 to 1768 patients). Overall, 1897 (19%) patients had deep vein thrombosis, and there were study differences in case mix and deep vein thrombosis prevalence. On average across the 12 studies, the overall calibration was excellent, with a summary E/O of 1.02 (95% confidence interval 0.79 to 1.32), revealing that total number of predicted and true cases of deep vein thrombosis was almost in perfect agreement (that is, an E/O close to 1). However, a random effects meta-analysis revealed considerable between-study heterogeneity. The I^2^ statistic was 97%, which indicated that 97% of the total variation in the study estimates was due to between-study heterogeneity. The large heterogeneity is also evident in the forest plot (fig 2), with large variation in study estimates and many non-overlapping confidence intervals. The summary E/O estimate was therefore an incomplete picture, because performance in particular populations could vary considerably from the average.

Rather than focusing on I^2^, which might be misleading when the study sample sizes are large,^55^ the extent of heterogeneity in model performance is better quantified by a 95% prediction interval.^52^ Debray and colleagues calculated an approximate 95% prediction interval for E/O in a new population, which was wide (0.38 to 2.72), indicating potentially large under-prediction (that is, E/O<1) or over-prediction (that is, E/O>1) of risk of deep vein thrombosis in some populations, a finding that was masked by focusing solely on the excellent summary performance. 

Similarly, the approximate 95% prediction interval for the C statistic was 0.64 to 0.73, indicating heterogeneous (and often only moderate) discrimination performance. The model was therefore deemed inadequate: it requires improvements (eg, recalibration or additional predictors) to reduce heterogeneity and improve discrimination to be clinically useful toward an accurate diagnosis of deep vein thrombosis. Indeed, other models for diagnosis of the disorder containing more predictors already exist, and appear to perform well across different subgroups and settings.^56^

### Example 2: Examining consistency in performance across multiple practices

Given big datasets from e-health records or disease registries, external validation can also use meta-analysis techniques to examine heterogeneity in model performance across different clusters—such as practices, hospitals, or countries where case mix and outcome prevalence (incidence) are likely to vary. Indeed, each cluster might be viewed as a different external validation study. For example, we extended Collins and Altman’s external validation of QRISK2 using data from 364 general practices,^44^ by performing a random effects meta-analysis to summarise the C statistic. The summary (average) C statistic was 0.83 (95% confidence interval 0.826 to 0.833). However, there was high between-practice heterogeneity in the C statistic (I^2^=80.9%) and the approximate 95% prediction interval for the true C statistic in a new practice was wide (0.76 to 0.88), although containing values that would typically be considered moderate or high discrimination.

Following such a meta-analysis, the use of forest plots to display cluster specific and meta-analysis results is often impractical given the number of clusters, such as the hundreds of practices observed within e-health records (such as the Clinical Practice Research Datalink (CPRD) and The Health Improvement Network (THIN)). A useful alternative approach to visualise any variability in model performance at the cluster level is to present plots of performance estimates versus their precision (or sample size). 

**Fig 3 f3:**
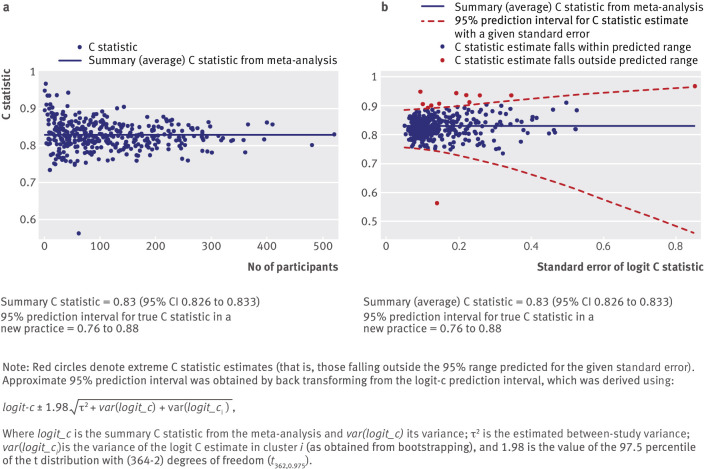
Funnel plots of discrimination performance (as measured by the C statistic) of QRISK2, across all 364 general practice surgeries in the external validation dataset of Collins and Altman.^44^ Plots show C statistic versus (a) number of cardiovascular events and (b) standard error of logit C statistic

Figure 3 shows a plot of the C statistic for QRISK2, for each of the 364 included general practices, versus either the number of outcome events in the practice or the standard error of the C statistic on the scale used in the meta-analysis.^57^ Such plots are often called funnel plots, and indeed in figure 3a[Fig f3] the distinctive funnel shape is reasonably well observed, where small practices (in this instance, defined on the x axis by the number of outcome events) show a wider variation in the C statistic than larger clusters. The extremes of the funnel help reveal particular general practices where the model is performing much better, or much worse, than on average. 

A formal statistical way to identify practices with extreme predictive performance is shown in figure 3b[Fig f3], where an approximate 95% interval is added to reveal where C statistic estimates are predicted to lie, given the standard error observed. Those points (in red) denote practices that fall outside the predicted range, with those below the lower boundary of particular interest. Of course, as this is a 95% interval, by definition we expect 5% of all practices to fall out of the region by chance. Nevertheless, we find it a helpful approach to identify, from hundreds of practices, those practices worthy of extra attention. In particular, it motivates enquiry to identify any striking reasons (aside from the play of chance) why the model performs so differently in these practices.

### Example 3: Examining performance in clinically relevant subgroups

Just as stratified medicine research examines whether a treatment works better or worse for some subgroups than others,^15^ the use of big datasets allows prediction model research to examine whether a model is more accurate for some subgroups than others. For example, the performance of QRISK2 has been examined in different ethnic groups^58^ ^59^ and in patients with diabetes.^60^ The examination of patients with diabetes was conducted in response to a recommendation by the National Institute for Health and Care Excellence to not use QRISK2 in patients with type 1 or 2 diabetes.

**Fig 4 f4:**
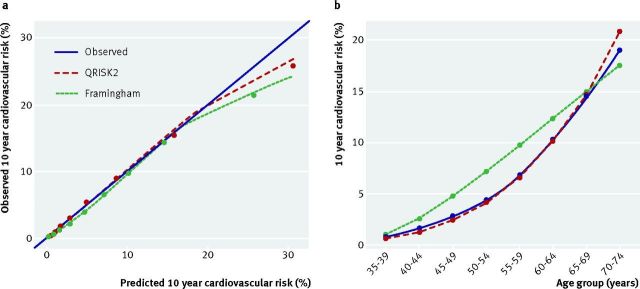
Calibration of QRISK2 and the Framingham risk score in women aged 35 to 74 years, (a) by tenth of predicted risk augmented with a smoothed calibration curve, and (b) within eight age groups. Dotted lines=denote perfect calibration

The recent TRIPOD guideline^34^ ^35^ also indicates that a model’s predictive performance should be evaluated in relation to key variables, such as age or sex subgroups, rather than just across all individuals combined, which can mask any deficiencies in the model. For example, an external validation study of QRISK2 and the Framingham risk score assessed model calibration both in the entire cohort (by each tenth of predicted risk) but also by age groups.^44^ Over the entire sample of 1.1 million women in the cohort (from the THIN database), both models showed good overall calibration between predicted and observed 10 year cardiovascular risk, with an E/O of 1.01 for QRISK2 and 1.03 for the Framingham risk score. This is illustrated in figure 4a[Fig f4], although there is slight over-prediction observed in women at higher 10 year cardiovascular risk, which is more pronounced for the Framingham risk score.

The big datasets enable further interrogation of predictive performance, for example, by five year age groups (fig 4b[Fig f4]). It is immediately apparent that Framingham over-predicts the 10 year cardiovascular risk in women aged 40 to 64 years and under-predicts risk in women aged 70 to 74 years (fig 4b[Fig f4]). By contrast, QRISK2 seems to accurately predict 10 year cardiovascular risk across all age groups. This was not revealed by the summary calibration plot typically used (fig 4a[Fig f4]). Further work could also examine between-practice heterogeneity in the calibration performance for each age group, and similarly look at performance within categories of other important subgroups (eg, ethnicity).

### Example 4: Examining sources of heterogeneity in model performance

Where model performance is heterogeneous, the sources of heterogeneity can be investigated. For example, Pennells and colleagues^30^ used IPD from multiple studies to evaluate a prediction model for coronary heart disease, and showed (using meta-regression) that its discrimination performance improved in studies with a larger standard deviation of age. Every five year increase in standard deviation improved the C statistic by about 0.05. Thus, larger case mix variation (measured here by the variability of age in each population) is related to larger discrimination performance; in other words, populations with a narrower case mix (more homogeneous predictor values across individuals) tend to have worse discrimination performance. 

**Fig 5 f5:**
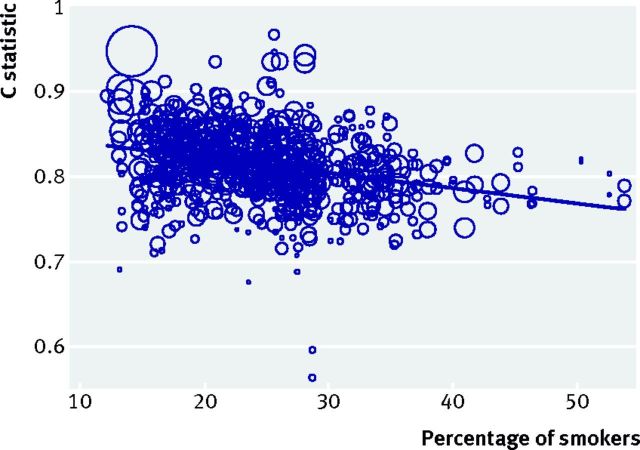
Association between percentage of smokers and C statistic for QRISK2 across all 364 general practice surgeries in the external validation dataset of Collins and Altman.^44^ Circle size is weighted by the precision of the C statistic estimate (that is, larger circles indicate C statistic estimates with smaller standard errors, and thus more weight in the meta-regression). Note: the solid line shows the meta-regression slope when data are analysed on the C statistic scale; similar findings and trends were obtained when reanalysing the logit C statistic scale

We further extended our investigation of QRISK2, and found that the C statistic decreases across practices as the population’s mean age and percentage of smokers increase (fig 5). This suggests that discrimination as measured by the C statistic is lower in populations with a higher risk of cardiovascular disease, which again could be due to narrower case mix variation, but could alternatively (or additionally) be due to differences in the magnitude of predictor effects in such populations. This is now subject to further research.

### Example 5: Examining model recalibration strategies (model updating)

**Fig 6 f6:**
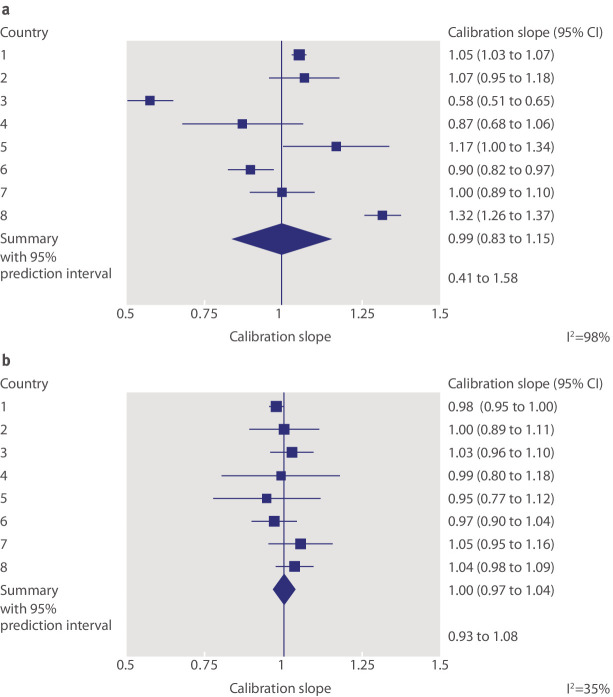
Calibration performance (as measured by the calibration slope) of the breast cancer model evaluated by Snell and colleagues^53^ before and after recalibration of the baseline mortality rate in each country. (a) Forest plot assuming the same baseline hazard rate in each country (no recalibration). (b) Forest plot allowing a different baseline hazard rate for each country (recalibration)

Snell and colleagues^53^ used IPD from eight countries to externally validate a prediction model of mortality risk over time in patients with breast cancer. They identified large between-country heterogeneity in calibration performance, as shown by a wide 95% prediction interval for the calibration slope (0.41 to 1.58; fig 6a[Fig f6]). This signals potential differences in the baseline mortality rates across countries, or differences in the effects of included predictors. It is also possible that important predictors (such as interactions and non-linear effects) are missing from the model that would otherwise explain such differences.

In such situations, researchers might be tempted to discard the model entirely but this is premature, because performance can often be improved if (simple) recalibration strategies are allowed.^20^ Recalibration is a form of model updating, where particular components of the developed model (such as the intercept or baseline hazard rate, or even particular predictor effects) are modified or tailored for each study population of interest. For instance, Snell and colleagues extend their work by examining whether the model’s calibration performance improves with recalibration of the baseline hazard function in each country. So although the model’s predictor effects were not modified, the baseline hazard of the developed model was re-estimated for each country to enhance risk predictions. This is akin to diagnostic test research, where post-test probabilities are best tailored to the disease prevalence of the population at hand.^61^ ^62^ ^63^ There was a dramatic improvement in the breast cancer model performance (fig 6b[Fig f6]): I^2^ fell from 98% without recalibration to 35% with recalibration, and the updated 95% prediction interval for the calibration slope was 0.93 to 1.08, which is now narrow and close to 1. The importance of baseline risk recalibration is also shown elsewhere.^51^ ^64^

## Practical and methodological challenges

Although the availability of big datasets offers many opportunities for external validation research, potential methodological challenges also arise.^28^ ^29^ ^65^ In particular, missing predictor values are likely in some participants and there may be systematically missing predictors, which occurs when a predictor is not measured for any individuals in one or more studies (clusters). Advanced multiple imputation techniques are then necessary (under a missing at random assumption),^66^ ^67^ otherwise the prediction model cannot be validated in the clusters with missing predictors. Further, although exploration of heterogeneity in model performance is an opportunity, the potential causes of heterogeneity should ideally be specified in advance, to avoid data dredging and spurious (chance) findings.

The quality of e-health records is of particular concern, because they contain data routinely collected that might not be as rigorous as the IPD from a meta-analysis of research studies. A dataset being large does not imply it is of high quality; indeed, the opposite may be true. In relation to CPRD, Herrett and colleagues^68^ state: “The quality of primary care data is variable because data are entered by GPs [general practitioners] during routine consultations, not for the purpose of research. Researchers must therefore undertake comprehensive data quality checks before undertaking a study.” Among others, particular weaknesses include:

- Missing data (and its potential to be missing not at random)
- Non-standardised definitions of diagnoses and outcomes
- The need to interpret an absence of a “read code”’ for a disease or outcome as absence of the disease or outcome itself, when sometimes patients with the disease or outcome simply fail to present to the general practitioner
- Incomplete follow-up times and event dates (such as hospital admission and length of stay)
- Lack of recording of potentially important and novel predictors.

Thus, just as IPD meta-analyses should examine the risk of bias of included studies,^69^ researchers using e-health or routine care registry databases should examine the quality of their data.

Research using big datasets can also be expensive. For example, according to the general terms and conditions on the CPRD website (https://www.cprd.com/dataAccess/) for “the sum £255,000 per annum the Licensor grants the Licensee a limited, non-exclusive and non-transferable licence on the terms of this Licence for up to 2 Nominated Users to access the Services.” Costs are reduced for certain parties, for example, at about £130 000 ($187 500; €228 400) per year for academia in our experience. The use of large data from established cohorts (such as UK Biobank) is an alternative and much cheaper option; for example, according to their website (www.ukbiobank.ac.uk/scientists-3/), access to UK Biobank data costs “£1,500+VAT (where applicable) per application that requires access to data only”. However, such cohorts often have a narrower case mix than the wider population, due to specific entry criteria; for example, UK Biobank recruited individuals aged between 40 and 69 years. 

For IPD meta-analysis situations, it can also be expensive, time consuming, and generally painstaking to obtain and clean the raw data from multiple studies.^70^ Further, not all desired studies may provide their IPD, and the available IPD might be from a selective, non-random part of the evidence base.^71^ Another challenge to the use of IPD from multiple studies—or multiple e-health or registry datasets—is how to identify and deal with individuals who contribute data to more than one dataset.

Researchers might also want to use the large dataset to both develop and externally validate a model. Thus, they need to decide whether and how a subset of the data is excluded for the validation phase. Big datasets from e-health records often contains hundreds of clusters and thousands of participants and events; in such situations, a sensible approach is to omit 20 or more clusters for external validation, which are chosen in advance (non-random sample) to cover a wide range of different populations, settings, and case mix variations. 

In an IPD meta-analysis, where the number of studies (*k*) is typically fewer than 10 studies, a process known as internal-external cross validation has been proposed to combine model development with validation.^42^ ^45^ Here, all but one of the studies are used for model development, with the remaining study used for external validation. This process is repeated a further *k*−1 times, on each occasion omitting a different study to ascertain external validation performance. If performance is always adequate, a final model may be developed using all studies. Otherwise, it flags heterogeneous study populations where a developed model might not perform well, and signals that model updating strategies might be needed (such as recalibration). We note, however, that each cycle should ensure an adequate sample size for model development^72^ ^73^ ^74^ and the use of appropriate model derivation techniques (eg, adjustment for optimism).^16^ ^26^ Otherwise, poor performance could simply reflect small sample sizes, overfitting, and substandard development techniques.

For model development, the use of big datasets could lead to many candidate predictors being statistically significant, even when they only improve prediction by a small amount. Therefore, a more considered process of predictor selection (eg, based on clinical relevance and magnitude of effect, not just statistical significance) will be required to avoid inclusion of a vast number of predictors unnecessarily. It might also be helpful to ascertain which candidate predictors are heterogeneous across clusters, to limit eventual heterogeneity in model performance; Wynants and colleagues suggest the residual intraclass correlation for this purpose.^75^ Further details of the methodological challenges facing IPD meta-analysis of prognosis research are given elsewhere.^28^ ^65^

## Reporting of external validation studies that use big datasets

Box 2 provides some initial suggestions to extend the recent TRIPOD statement for reporting external validation studies that use big datasets.^34^ ^35^ Ideally, these should be refined and potentially extended in an international consensus process, and work on this has begun by the TRIPOD initiative. Our aim with box 2 is to provide some interim guidance for researchers, which also draw on the recent PRISMA-IPD guidelines.^76^ Graphical displays presenting model performance are particularly important. In particular, forest and funnel plots can be used to display meta-analyses as shown above, ideally with calibration plots for the whole dataset and in each cluster separately, as shown elsewhere.^42^ ^45^

**Box 2: Suggested initial extensions to the TRIPOD guidelines^34^ ^35^ for the reporting of external validation studies that use big datasets (such as those generated from IPD meta-analysis or e-health databases)**

**How data were obtained**

When using data from multiple studies, describe:
  - How the studies were identified (eg, systematic review, collaborative project of selected researchers)
  - Which studies were approached for their data, and how (eg, email, letter)
  - The proportion of identified studies that agreed to provide their data, and the design of these studies (eg, randomised trials, cohort studies, cross sectional studies)
  - Whether studies providing IPD were similar (eg, in terms of their populations, design) to studies without IPD.
When using data from e-health records, describe the process toward obtaining the data and whether multiple databases were used (for example, for linkage of predictor and outcome information).

**Clustering in the data**

Summarise the clustering in the data (eg, due to practices, hospitals, studies) and the different populations each cluster represents (eg, different regions, countries).
State the number of clusters in the entire dataset and the number of patients and events in each. If the number of clusters is large, then—for ease of presentation—the distribution of patient characteristics and events across clusters might be displayed by histograms and summary measures such as the mean, median, standard deviation, and minimum and maximum.
Report differences in case mix variation across clusters (eg, in the mean or standard deviation of predictor values), perhaps with a summary table or graphical display of baseline characteristics in each cluster.
Provide details of any other inconsistencies across clusters, for example, in the definition and measurement of predictors, the classification of the disease or outcome to be predicted, and the treatment strategies used.

**External validation analyses**

For each external validation analysis, state the numbers of patients, events, and clusters that were used.
Explain any methodological challenges in using or combining the data across clusters. In particular, state how any missing data were handled in each cluster (especially systematically missing predictors) and how any between-cluster differences in predictor or event definitions were handled.
Report the external validation performance in the whole dataset, including a weighted (meta-analysis) average across clusters, and in relation to clinically relevant subgroups or important variables.
Summarise the external validation performance in each cluster (eg, in a forest or funnel plot), and quantify the between-cluster heterogeneity in performance, for example, via a random-effects meta-analysis and deriving 95% prediction intervals for calibration and discrimination performance in a new cluster.
Explain any model updating (eg, recalibration) techniques examined, and report how average performance and heterogeneity in performance improves (or worsens) after updating.
Provide graphical displays to supplement the results, such as forest (or funnel) plots to display the meta-analyses, and calibration plots covering tenths of predicted risk and relevant subgroups, ideally for the whole dataset and in each cluster separately.

## Conclusions

We have highlighted how big datasets from multiple studies and e-health or registry databases provide novel opportunities for external validation of prediction models, which we hope will encourage researchers to interrogate the adequacy of prediction models more thoroughly. In particular, researchers should use their big datasets to check a model’s predictive performance (in terms of discrimination and calibration) across clinical settings, populations, and subgroups. Simply reporting a model’s overall performance (averaged across all clusters and individuals) is not sufficient because it can mask differences and important deficiencies across these clusters and subgroups. Potential users need to know whether a model is reliable or transportable to all the settings, populations, and groups represented in the data. 

If a model does not have consistent predictive performance, users must know the potential magnitude of the inaccuracy to make a better judgment of the model’s worth, and in whom. Further, users should be told when, and which type of, model updating or tailoring strategies (such as recalibration) are necessary for particular settings or clusters, and by how much they improve predictive performance.^20^ We demonstrated these issues using empirical examples. Sometimes, even with updating or tailoring strategies, a model may not be transportable to particular settings, and an entirely new model might be required. For example, a model that was developed from practices containing predominately one ethic group are unlikely to perform as well in the wider population of the United Kingdom if there is heterogeneity in predictor effects and baseline risks across different ethnic groups. In such situations, important predictors are missing from the model. An example is the Cambridge diabetes risk score, which was developed from practices in predominately white population areas of the UK, and does not discriminate as well as the QDS score (now known as QDiabetes), which was developed on a wider set of ethnically diverse practices.^77^

Our work agrees with Van Calster and colleagues,^37^ who encourage researchers to examine a model’s calibration performance to a higher level. They state that “a flexible assessment of calibration in small validation datasets is problematic,” but our examples show how big datasets can help deal with this. Other issues might also benefit from big datasets, such as comparing (and even combining^78^) multiple competing models,^79^ and examining the added value of a new predictor,^30^ for example, in terms of the net benefit for clinical decision making.^80^ A full discussion of the different research questions one may address in big datasets, such as an IPD meta-analysis, for clinical prediction model research is given by Debray and colleagues.^29^

In conclusion, access to big datasets from, for example, e-health records, registry databases, and IPD meta-analyses should signal a new approach to external validation studies in risk prediction research, for either diagnostic or prognostic purposes. Recent articles in *The BMJ* call for data sharing to be “the expected norm,”^81^ and for synthesis of IPD to have greater impact on clinical guidelines.^82^ Our examples reinforce why such calls are of utmost relevance for the validation of prediction models, as we strive to ensure developed models are reliable and fit for purpose in all the settings of intended use.

## Notes

We thank the editors and reviewers from *The BMJ* for their constructive feedback and suggestions on this paper, which helped improve the article on revision.

Provenance and peer review: Not commissioned; externally peer reviewed.
