# External validation of clinical prediction models using big datasets from e-health records or IPD meta-analysis: opportunities and challenges

**DOI:** 10.1136/bmj.l4379

**Published:** 2019-06-25

In this article by Riley and colleagues (*BMJ* 2016;353, doi:10.1136/bmj.i3140, published 22 June 2016 and in the 25 June 2016 print issue), a reader pointed a mistake in the labelling of figures 3 and 6 (https://twitter.com/bogdienache/status/1139178312289411072). The online article and PDF have since been updated with corrected figures. The authors would like to thank the reader for drawing this to their attention.

